# Risk Factors Associated With Bell's Palsy: A Real‐World Analysis of 281,600 Patients

**DOI:** 10.1111/ene.70336

**Published:** 2025-08-21

**Authors:** Norbert Neckel, Susanne Nahles, Max Heiland, Heinrich Audebert, Anna Zdunczyk, Orlando Guntinas‐Lichius, Robert Preissner, Saskia Preissner

**Affiliations:** ^1^ Department of Oral and Maxillofacial Surgery, Campus Virchow‐Klinikum Charité – Universitätsmedizin Berlin, Corporate Member of Freie Universität Berlin and Humboldt‐Universität Zu Berlin Berlin Germany; ^2^ Department of Neurology and Center for Stroke Research Charité – Universitätsmedizin Berlin, Corporate Member of Freie Universität Berlin and Humboldt‐Universität Zu Berlin Berlin Germany; ^3^ Department of Neurosurgery Charité – Universitätsmedizin Berlin, Corporate Member of Freie Universität Berlin and Humboldt‐Universität Zu Berlin Berlin Germany; ^4^ Department of Otorhinolaryngology Jena University Hospital Jena Germany; ^5^ Institute of Physiology and Science‐IT Charité Universitätsmedizin Berlin, Corporate Member of Freie Universität Berlin, Humboldt‐Universität Zu Berlin and Berlin Institute of Health Berlin Germany

**Keywords:** Bell's palsy, depression, herpes, pregnancy, risk factors

## Abstract

**Introduction:**

Bell's palsy is the most common cause of peripheral facial paralysis, with an annual incidence of 5–50 per 100,000 cases worldwide. Its etiology remains largely unknown, though risk factors such as herpes simplex virus reactivation, diabetes, depression, and pregnancy‐related complications have been suggested. Understanding these risk factors is critical for improving diagnosis, prevention, and treatment strategies.

**Methods:**

A retrospective analysis of the TriNetX database included over 25 million patients. Two cohorts of approximately 140,800 patients each, matched for age and sex, were analyzed for associations between BP and herpes simplex virus, diabetes, depression, and pregnancy. Odds ratios (OR) and confidence intervals (CI) were calculated, with *p* < 0.05 indicating significance.

**Results:**

Herpes simplex virus showed the strongest association with Bell's palsy (OR: 6.49, 95% CI: 5.96–7.05); followed by diabetes (OR: 2.4, CI: 2.36–2.46) and depression (OR: 2.05, CI: 2.0–2.1). Pregnancy was inversely correlated (OR: 0.76, CI: 0.73–0.78).

**Conclusion:**

Herpes simplex virus reactivation appears to be a major risk factor, suggesting a potential role of antiviral therapies in select cases. The associations with diabetes and depression highlight a need for metabolic and mental health evaluations in patients with Bell's palsy. The inverse correlation with pregnancy warrants further investigation into pregnancy‐related conditions. These findings emphasize the multifactorial nature of this condition and the importance of individualized approaches to reduce its idiopathic classification.

## Graphical abstract

This study analyzed over 280,000 patient records to identify key risk factors associated with Bell's palsy. The strongest association was found with herpes simplex virus reactivation, followed by diabetes and depression, while pregnancy showed an inverse correlation. These findings highlight the multifactorial nature of Bell's palsy and support more individualized diagnostic and treatment approaches.

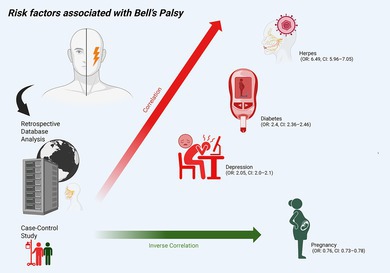

**Abbreviations**
BP: Bell's palsy
CI: confidence interval (Konfidenzintervall)
HCO: health care organization
HSV: herpes simplex virus
ICD‐10: International Classification of Diseases, 10th Revision
OR: odds ratio
QoL: quality of life (Lebensqualität)
VZV: varicella zoster virus

## 1 Introduction

Even though there have been reports of bilateral cases in the past, Bell's palsy (BP) is generally defined as a sudden, idiopathic, unilateral facial nerve dysfunction, typically peaking within 48–72 h [1, 2, 3]. It is the most common cause of peripheral facial paralysis, accounting for 60%–75% of such cases and therefore the majority of facial palsy cases are “of unknown cause” [1, 2]. The condition manifests with the inability to close the eye, drooping of the mouth, and flattening of the nasolabial fold, often accompanied by periauricular pain, taste disturbances, hyperacusis and dry eyes due to reduced lacrimation [1, 2, 4]. 70%–85% of the patients recover fully, but outcomes of BP vary, depending on timing and type of treatment, individual patient characteristics and comorbidities [1, 2, 5, 6, 7]. Partial loss of function is associated with better chances for recovery [7]. 13% of the cases recover only partially, but roughly 16% develop long standing paralysis with severe loss of function and synkinesis [1, 7]. Regional incidence rates vary significantly ranging from 5 to 50 per 100,000 cases [5, 6, 8, 9]. The lifetime risk is quantified as 1 out of 60, resulting in a relevant health care burden [9, 10]. Despite this, the exact etiology remains elusive, but it is thought to be associated with certain risk factors, such as the (undetected) reactivation of latent herpes simplex virus infection, leading to inflammation and swelling of the facial nerve [1, 4, 5]. International guidelines primarily recommend oral corticosteroids, while antiviral medications may have additional beneficial effects [7, 11, 12, 13, 14, 15]. Early nonmedical care focuses on eye protection, with facial reanimation procedures (e.g., nerve grafting, neurotized free tissue transfer) considered later [4, 7, 16, 17, 18]. In slowly progressing cases without improvement of the paralysis surgical exploration with biopsy of the nerve can be warranted [19]. The data on physical therapy and nerve decompression are too heterogenous for clear recommendations [7]. BP by definition rules out any other tangible diagnosis, such as traumatic, inflammatory, infectious, neoplastic or systemic causes and it can be hardto distinguish between risk factors and mechanisms, especially in the case of viral infection or diabetic neuropathy [7, 20].

Other factors are hypertension, dyslipidemia, obesity, pregnancy, immunogenic dysregulation, genetics, as well as sociodemographic factors such as age, gender, consanguineous parents [6, 8, 20, 21, 22]. The abundance of potential influencing factors suggests a multifactorial genesis. This is underscored by the variability in incidence rates across different regions and populations [6, 20].

Consequently, this may explain the inconsistency in existing studies regarding the relevance of pregnancy, age, and gender, requiring large sample sizes to reduce the risk of overfitting and allow the identification of actual risk factors rather than random noise [6, 8, 20, 23].

Therefore, this study aimed to challenge the previous reports and to uncover new risks or protective factors for BP using a real‐world approach.

## 2 Methods

The TriNetX database is a collaborative research network providing medical data of over 100 health care organizations (HCOs) worldwide [24]. Due to the de‐identification of the data through the TriNetX database, this retrospective research was declared as nonhuman subject research by the Institutional Review Board.

The database was accessed on 24th of September 2024 and searched for inpatients with and without the diagnosis of BP (International Classification of Diseases Code (ICD‐10): G.51.0). To evaluate common risk factors, we defined the following diagnoses before or within 30 days after the index event (diagnosis of BP): herpes simplex infections (ICD‐10: B00) or herpes zoster (ICD‐10: B02), pregnancy, childbirth, and puerperium (ICD‐10: O00‐O9A), diabetes (ICD‐10: E08‐E13), and depression (ICD‐10: F32). Patients were matched for age and sex using 1:1 propensity score matching. Risk analyses were performed calculating risk differences, risk ratios (RR) and odds ratios (OR). Statistical analyses utilized the Log‐Rank test, with the threshold for statistical significance set at 5% (*p* = 0.05). Due to the low number of tests, no post hoc analysis was performed.

### 2.1 Eligibility Criteria

Patients were included if they had a documented inpatient encounter and an index event within the last 20 years. Cohort 1 (+bell) included patients with a diagnosis of Bell's palsy. Cohort 2 (−bell) included inpatients without any diagnosis of Bell's palsy (G51.0); these patients were explicitly excluded if they had ever received such a diagnosis. Only patients with complete demographic data and qualifying index events were included. Diagnoses occurring more than 30 days after the index event were not considered in the outcome analysis.

## 3 Results

**FIGURE 1:**
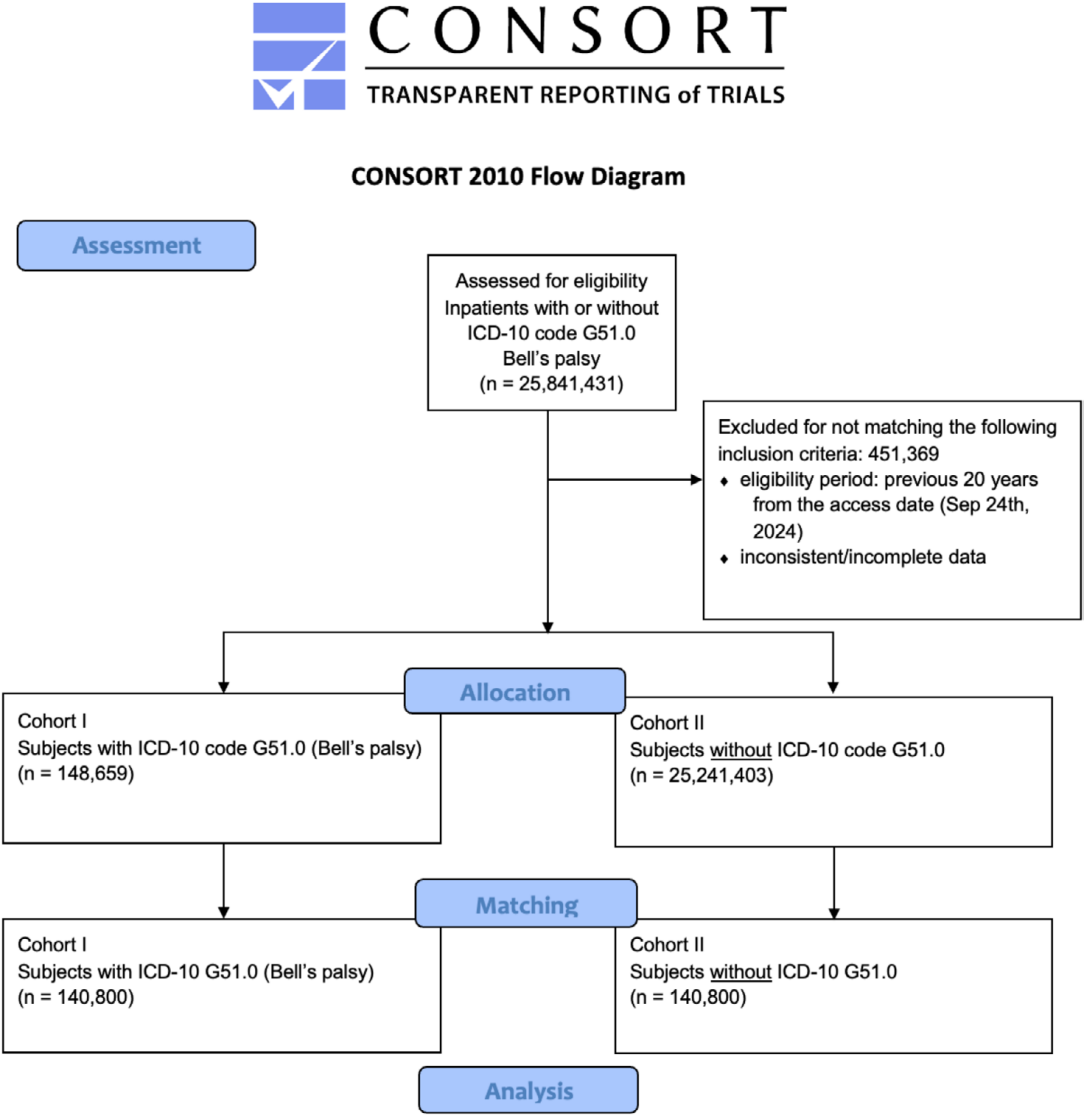
Modified CONSORT diagram for the performed analysis.

About 80 HCOs answered with over 25 million patients for the two cohorts (Figure 1).

Propensity score matching resulted in two homogenous cohorts of about 140,800 patients in each cohort, with a mean age ± standard deviation of 55.1 ± 20.7 years and 53% females.

**FIGURE 2:**
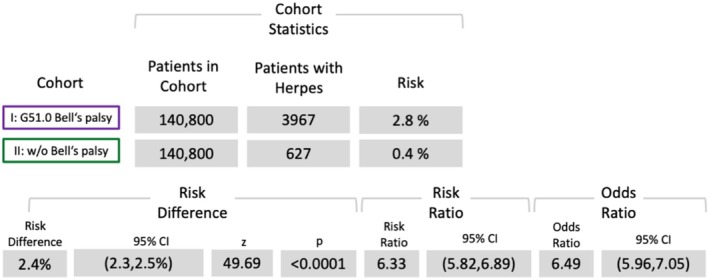
Results of the risk analysis for herpes simplex, herpes zoster (ICD‐10: B00 or B02).

**FIGURE 3:**
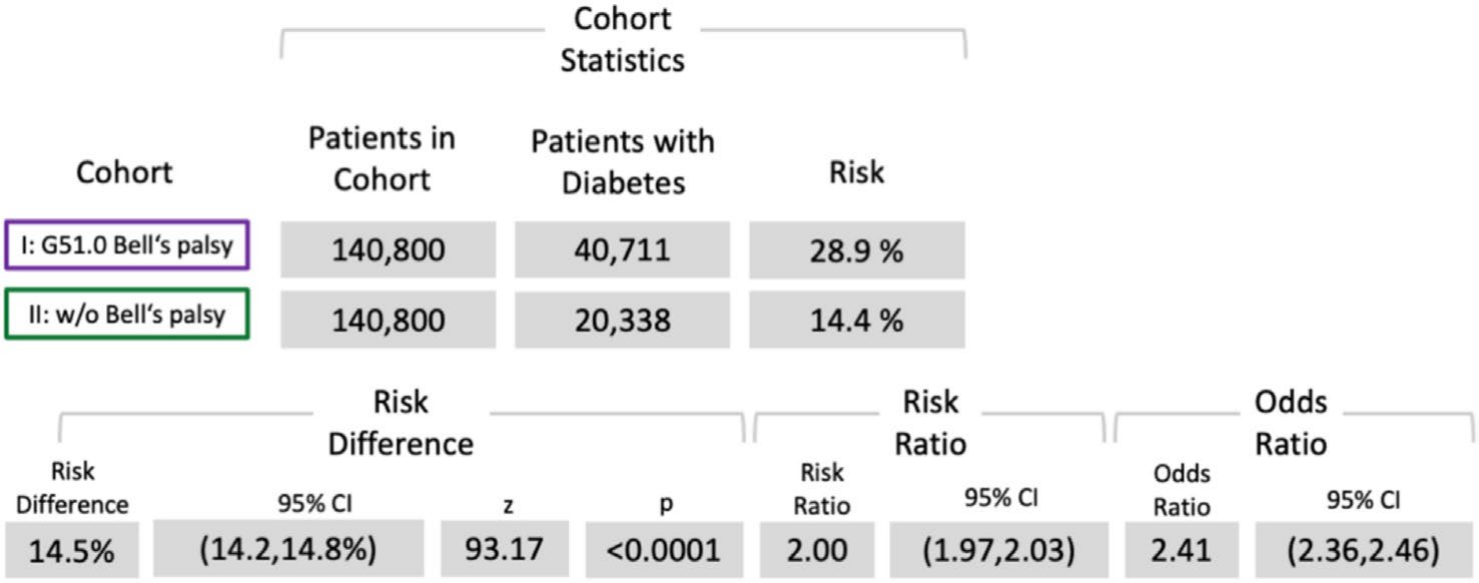
Results of the risk analysis for diabetes (ICD‐10: E08‐E13).

**FIGURE 4:**
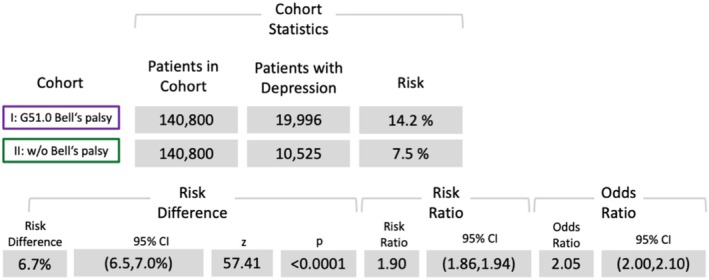
Results of the risk analysis for depression (ICD‐10: F32).

**FIGURE 5:**
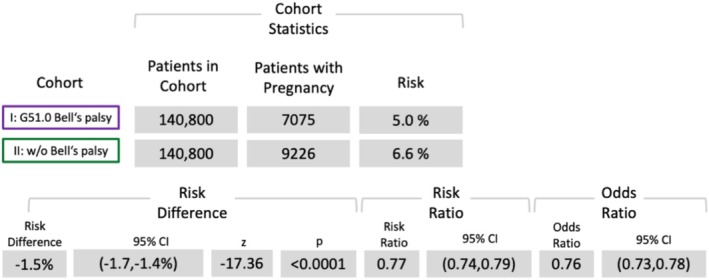
Results of the risk analysis for pregnancy, childbirth, and puerperium (ICD‐10: O00‐O9A).

Risk analyses revealed significantly elevated risks for herpes (OR (95% confidence interval [CI]): 6.49 [5.96, 70.5]) (Figure 2), diabetes (OR: 2.4 [CI: 2.36, 2.46]) (Figure 3), and depression (OR: 2.05 [CI: 2.0, 2.1]) (Figure 4). However, the risk analysis yielded a significant negative correlation of BP for pregnancy (OR: 0.76 [CI: 0.73, 0.78]) (Figure 5).

## 4 Discussion

In this database analysis, herpes infection demonstrated the strongest association with BP, followed by diabetes and depression. In contrast, pregnancy was inversely correlated. These findings provide new insights into the multifactorial etiology of BP.

Depression may relate to both psychological and biological mechanisms in BP. Facial appearance plays a key role in social interaction, and facial asymmetry due to BP can significantly affect perceived quality of life (QoL) and self‐esteem, especially in chronic cases [25, 26]. Humans are able to rapidly extract age, gender, and emotional state from faces, allowing for swift judgments about personality, health, and attractiveness [25, 27]. Consequently, even minor esthetic modifications to this anatomically significant area including BP can have a substantial impact on perceived quality of life (QoL) [28, 29]. No wonder, BP significantly impacts QoL, affecting overall well‐being and is strongly associated with the social and psychosocial scoring domains. This is especially the case for longer standing paralysis and could be explained by an augmenting effect in social interaction over time [29]. The significant correlation of the diagnosis of depression with BP might be attributed to these detrimental psychological effects of BP. However, in this study only preexisting or newly diagnosed depression was included. A similar correlation with preexisting depression could be found in a longitudinal nationwide health insurance database study in Taiwan, which used a dataset of a 17‐year period including 82,662 patients [30]. The authors were able to establish a clear temporal relationship between depression and consecutive BP in the cohort of 20–64 years old bearing a 1.32‐fold increased risk [30]. One common feature of both conditions could be some degree of immunomodulation, but a conclusive link or the precise mechanisms remain unknown [30, 31]. However, psychoimmunological effects were shown to exist and influence health and the course of a disease [32, 33].

Since the evidence in that matter is still quite limited and controversial, further studies addressing this issue are urgently needed [30, 34]. However, not adjusting for the temporal sequence might therefore lead to misguided conclusions [30].

Despite its clinical and socioeconomic burden, BP remains poorly understood. Identifying associated factors such as viral infections, metabolic disorders, or psychological comorbidities is essential to reduce its idiopathic classification and improve prevention and treatment strategies [29]. This results in comparatively unspecific therapeutic approaches, of which some also lack substantial evidence [7, 14, 35]. Therefore, identification of such risk factors and/or causative elements is pivotal for their prevention and treatment. In cases without recovery, surgical procedures are proposed to reanimate the paralyzed face [16, 17, 18].

Potential clinical etiologies include anatomical factors, virus infections, ischemia, and immunogenic or inflammatory causes [31]. Even though evidence exists for all of these potential influencing factors, none could be singled out as the driving cause, leading the authors of a recent review to categorize these potential risk factors as explanatory theories [31]. Potentially, BP consists of a variety of underlying pathophysiological mechanisms that yet need to be uncovered.

In the present study, anatomical factors could not be evaluated. Since variations of the internal auditory canal in shape and diameter, which are considered to be potentially responsible for BP, are usually not coded separately in the healthcare system [31, 36]. The sudden onset of facial palsy argues against these anatomical variations as a primary cause rather than a predisposing factor if swelling of the facial nerve or increase in diameter occurs.

This can be caused by external and internal factors, including the aforementioned theories. Virus infections, specifically by herpes simplex virus (HSV) and varicella zoster (VZV) are known to target peripheral neurons and can reside for a lifetime in cranial and neck ganglia [31]. Reactivation is thought to cause nerve damage through demyelination and neurapraxia, which may trigger Wallerian degeneration and result in permanent facial palsy [9, 37]. Evidence exists for HSV‐1, with BP being linked to the presence of virus DNA in the endoneurial fluid of the affected facial nerves [38]. In animal models, it can also cause facial palsy and be reactivated by immunomodulation [31, 39]. This is in line with the findings of this databank analysis. The OR of roughly 6.5 in this context strongly supports the hypothesis of an association between herpes and BP.

This highlights the importance of herpes viruses as a risk factor for facial paralysis and suggests that a significant number of BP cases may be caused by otherwise undetected or unassociated viral reactivation. The identification of these cases is of particular interest as the use of antiviral therapies is not well established and is not generally recommended [7, 11, 35]. However, the presented data suggest that a certain cohort of patients might benefit from it.

Another widely recognized potential risk factor is local ischemia [31]. While many different sources for peripheral neuropathy exist, diabetes is the most common reason [40]. Bosco et al. [41] even suggested that BP might be considered a hint to prediabetes, demonstrating that the prevalence of pathologic glucose metabolism was significantly higher in patients with BP. Furthermore, Mueanchoo et al. [42] could show that increased cardiometabolic risk and especially elevated free blood sugar were associated with BP. In patients with normal blood glucose levels, they found a significant correlation with impaired 2‐h oral glucose tolerance. Just recently, Fann et al. [30] found that in younger patients of < 40 years of age, diabetes was an independent predictor of BP. In line with these findings, our data demonstrate a significant correlation of the diagnosis of diabetes with a concomitant BP. However, this correlation was not as strong as with herpes infections. Nevertheless, BP patients should undergo thorough screening for glucose metabolism disorders and other cardiometabolic risks like high blood pressure and metabolic syndrome, which are also associated with a higher risk of BP.

Although the overall prevalence of BP is similar in men and women, BP has repeatedly been associated with pregnancy [9, 10]. Recent publications highlight the limitation of data and therefore the evidence available addressing this topic, while some emphasize that pregnancy was associated with worse prognosis [21, 43]. This is mostly based on the findings by Phillips et al. and Gillman et al., who demonstrated that pregnant women had worse long‐term facial function outcomes in the eFACE and were more likely to develop complete facial paralysis, respectively [21, 44, 45]. According to these authors, the cautious prescription of pharmacological treatments such as glucosteroids in pregnant women might be accountable [44, 45]. This assumption was challenged by Gaudin et al. [46], who found that even with steroid treatment, pregnant women had worse outcomes compared to nonpregnant controls. Another publication mentions recovery rates of 93% in pregnant women, though these findings are limited by a 30% dropout rate [21, 47]. In any case, it is striking that conditions coinciding with pregnancy, such as hypertension and diabetes, are significantly associated with BP in nonpregnant cohorts as well [21, 48]. This might explain why especially the third trimester, which is associated with the highest prevalence of these risk factors, seems to bear a higher risk for the development of BP [49]. Our data therefore suggest that pregnancy may not be an independent risk factor. However, certain consequences, side effects, or complications during pregnancy might be the reason for the inconsistent data found in the literature. Considering that aside from hormonal and metabolic changes, pregnancy is associated with a special immunological situation manifesting as periodontal disease [50]. As discussed previously in the context of depressive disorder, immunologic imbalance might be a potential factor in the development of BP.

Consequently, pregnancy‐associated diseases and risk factors need clinical and scientific consideration.

This study has several limitations. First, the use of a large patient database such as TriNetX may introduce unexpected associations and selection bias. Second, the temporal relationship between diagnoses could not be fully resolved, limiting the ability to distinguish cause from effect. It is also crucial to emphasize that the associations identified in this study should not be interpreted as evidence of causation. Additionally, the TriNetX database lacks information on the time interval between injury and treatment, which may introduce an unaccounted confounding variable. Finally, due to the retrospective design, only inpatients were included, possibly excluding milder outpatient cases and thereby introducing severity‐related selection bias. Despite these limitations, this study provides valuable real‐world data and supports the need for further prospective studies investigating specific risk factors, therapeutic approaches, and long‐term outcomes in Bell's palsy.

The goal must be to eradicate BP as a final diagnosis, as idiopathic can be equated with “not yet explainable”. Therefore, further and more detailed research on potential risk factors for disease incidence, treatment options, and recovery rate is needed to provide these specific patients with individualized and successful treatment options.

## 5 Conclusion

This real‐world data analysis identified strong associations between Bell's palsy and herpes infections, diabetes, and depression, while pregnancy showed an inverse correlation. Herpes simplex virus reactivation appears to be a major risk factor, suggesting a potential role for antiviral therapies in select cases. The associations with diabetes and depression highlight the need for metabolic and mental health evaluations in patients with Bell's palsy.

The inverse correlation with pregnancy contrasts with previous studies that have reported increased risk or worse outcomes in pregnant women. This discrepancy underscores the need for further investigation into pregnancy‐related conditions and their role in BP.

Overall, these findings emphasize the multifactorial nature of BP and highlight the importance of individualized diagnostic and therapeutic strategies to reduce its idiopathic classification and improve patient care.

## Author Contributions

N.N. and S.P. conceived the ideas, are responsible for the conceptualization, and wrote the initial draft of the manuscript. S.P. performed the statistical analysis. A.Z., H.A., and O.G.‐L. provided clinical context and helped interpret the findings. M.H., S.N., and R.P. provided the resources and supervised the project.

All authors contributed to the study by providing key insights, refining the methodology, and ensuring the accuracy of the analysis. All authors have critically reviewed the manuscript and approved the final version for publication.

## Ethics Statement

TriNetX, a global health research network, is committed to protecting healthcare data privacy and security. The network adheres to HIPAA and is ISO 27001 certified, ensuring strong data protection and compliance with the HIPAA Security Rule. All patient‐level data is de‐identified as per HIPAA standards, validated by expert determination, eliminating the need for an IRB waiver. Healthcare organizations provide data anonymously under a Business Associate Agreement, with shared data attenuated to prevent source identification. Patient consent is secured by HCOs, and research protocols follow ethical guidelines. These measures collectively uphold high standards of data privacy, security, and ethical governance within TriNetX. Therefore, no local ethics committee decision is required.

## Conflicts of Interest

The authors declare no conflicts of interest.

## Data Availability

Data from the TriNetX research network can be accessed via their platform, subject to a signed Data Use Agreement and the provision of de‐identified data only. Processing fees may apply. For more information or specific datasets, the corresponding author can be contacted upon reasonable request.
